# Mediterranean diet adherence, body composition, and recovery outcomes in hospitalised COVID-19 patients: a mixed-methods study

**DOI:** 10.3389/fnut.2026.1834540

**Published:** 2026-06-11

**Authors:** Kaja Teraž, Boštjan Šimunič, Rado Pišot, Saša Pišot

**Affiliations:** Institute for Kinesiology Research, Science and Research Centre Koper, Koper, Slovenia

**Keywords:** body composition, clinical nutrition, COVID-19 recovery, dietary patterns, lifestyle factors, Mediterranean diet, mixed-methods, rehabilitation

## Abstract

**Introduction:**

The COVID-19 pandemic highlighted the importance of nutritional and lifestyle factors in recovery from severe illness, yet their role in post-hospitalization rehabilitation remains insufficiently understood.

**Methods:**

This study investigated the association between adherence to the Mediterranean diet, body composition, and recovery-related outcomes in individuals following severe COVID-19 using an explanatory mixed-methods design. Thirty-nine patients were assessed at hospital discharge and after 8 weeks of unsupervised rehabilitation in a single-centre prospective cohort study conducted in Slovenia (ClinicalTrials.gov: NCT04860206, https://clinicaltrials.gov/study/NCT04860206).

**Results:**

Quantitative analyses showed a significant increase in fat-free mass over time and positive associations between fat-free mass and adherence to the Mediterranean diet. Qualitative findings further revealed that individuals with higher adherence demonstrated structured routines, adaptive coping strategies, and greater psychosocial resilience during recovery.

**Discussion:**

Together, these findings suggest that adherence to a Mediterranean dietary pattern, alongside related lifestyle behaviors, may support favorable body composition and enhance recovery processes following severe illness. This study provides an integrated perspective on physiological and behavioral determinants of recovery, highlighting the potential of nutrition-based approaches in post-COVID-19 rehabilitation.

## 1 Introduction

The Mediterranean diet, characterized by high consumption of fruits, vegetables, whole grains, legumes, nuts, olive oil, and moderate intake of fish and wine (1), has been extensively studied for its beneficial effects on health and longevity (2). Rooted in the traditional dietary patterns of countries bordering the Mediterranean Sea, this model is rich in antioxidants, anti-inflammatory compounds, and healthy fats, particularly monounsaturated fatty acids (2, 3). The Mediterranean diet’s emphasis on nutrient-dense, minimally processed foods provides essential vitamins, minerals, and phytonutrients that support metabolic functions and reduce oxidative stress (4). Healthy longevity is a multifactorial concept (5, 6) supported by scientific evidence, emphasizing the maintenance of physiological homeostasis—characterized by low systemic inflammation (7), optimal metabolic efficiency (8, 9), and a robust immune response (10)—alongside preserved cognitive function (11) and emotional well-being (12). In the context of this study, these dimensions are considered through recovery-related indicators following severe illness. Fundamental lifestyle factors, such as a balanced diet (13), regular physical exercise (14), and meaningful social interactions (15) collectively promote enduring health and functional autonomy in ageing populations. Physical activity not only helps manage body mass but also improves insulin sensitivity and cardiovascular health, among other health benefits. Numerous epidemiological studies and clinical trials have linked adherence to the Mediterranean diet with a reduced risk of chronic diseases, including cardiovascular disease, type 2 diabetes, and certain types of cancer (16–18). The Mediterranean diet and lifestyle are widely recognized for their positive effects on ageing populations, particularly in mitigating the risks associated with obesity and metabolic syndrome (17, 19). In the present study, recovery and long-term health is operationalized through recovery-related indicators, including body composition, dietary and lifestyle adherence, and psychosocial functioning, rather than long-term survival outcomes.

Obesity and excessive adipose tissue, significant precursors to metabolic syndrome, exacerbate the inflammatory activity of adipose tissue (20). This inflammation contributes to insulin resistance, dyslipidemia, and hypertension, all hallmark features of metabolic syndrome (20). Studies have demonstrated that adherence to the Mediterranean diet reduces levels of pro-inflammatory cytokines, such as IL-6 and TNF-*α*, while enhancing the production of anti-inflammatory adipokines, including adiponectin (21). Evidence suggests that adherence to the Mediterranean diet may reduce the risk, severity, and long-term consequences of COVID-19. Observational and clinical studies have shown that individuals with higher adherence to the Mediterranean diet had significantly lower serum levels of pro-inflammatory markers such as TNF-*α*, IL-1β, and hs-CRP, along with reduced oxidative stress (21–23). In patients with long COVID-19, greater adherence to the Mediterranean diet was associated with improved metabolic profiles, including lower BMI and uric acid levels and higher HDL cholesterol (24). The Mediterranean diet’s richness in antioxidants, monounsaturated fats, polyphenols, and essential micronutrients appears to modulate immune responses, reduce chronic inflammation, and support metabolic resilience—key factors in mitigating the severity and complications of COVID-19.

A Mendelian randomization analysis showed that elevated body fat mass, but not fat-free mass, causally increases the risk of severe COVID-19 or hospitalization (25). Higher relative fat mass—especially visceral and intermuscular adipose tissue—and lower fat-free mass are strong, independent predictors of severe COVID-19 outcomes (ICU admission, mechanical ventilation, mortality) (26). Conversely, adherence to a Mediterranean-style diet is linked to reductions in BMI and pro-inflammatory markers (IL6, TNFα, hs-CRP), which are recognized risk factors for poorer COVID-19 prognosis (22, 23, 27). The health-promoting effects of the Mediterranean diet cannot be fully understood without acknowledging the social and cultural practices that accompany it. In particular, promoting moderate physical activity and active social engagement plays a pivotal role in mitigating age-related physiological decline and enhancing overall quality of life (28). Moreover, the Mediterranean lifestyle further amplifies these benefits. Social engagement, a core integral aspect of Mediterranean culture, has been associated with reduced stress levels and enhanced mental well-being, factors that can indirectly support better metabolic health. Adherence to the Mediterranean diet and lifestyle—characterized by a high intake of plant-based foods, extra-virgin olive oil, fish, and regular engagement in physical and social activities—has been consistently associated with improved cardiometabolic profiles, attenuated systemic inflammation, reduced incidence of chronic diseases, and preservation of cognitive function (29). To further explore this relationship, associations between MEDLIFE index scores and qualitative data were examined. Collectively, these effects contribute to enhanced physiological resilience and improved recovery trajectories following illness. To address this gap, we investigated adherence to the Mediterranean diet in hospitalized COVID-19 patients and its association with body composition during recovery. Special attention was given to individuals with the highest adherence to specific components of the Mediterranean lifestyle. This study aimed to examine changes in body composition in relation to Mediterranean diet adherence and to integrate patients’ lived experiences to provide contextual insight into recovery processes.

## 2 Methods

### 2.1 Design and study sample

A single-center prospective cohort study was conducted, enrolling 43 consecutively hospitalized patients admitted for complications related to COVID-19. Functional and clinical parameters were assessed at hospital discharge (T1) and at a two-month follow-up after unsupervised rehabilitation (T2). Of the initial cohort, 39 participants completed both assessments.

Initially, 43 participants were included in the study, but 4 of them did not show up at T2, thus leaving a total of 39 patients tested on both periods (T1 and T2). Participants were recruited from patients hospitalized due to COVID-19 at the General Hospital Izola. The inclusion criteria for participating in the study were: age ≥ 18 years, a signed informed consent, and hospitalization due to COVID-19 disease (confirmed with a positive polymerase chain reaction (PCR) nasal swab test for SARS-CoV-2 virus). The exclusion criteria were a positive PCR test for COVID-19 virus at the time of hospital discharge and severe medical conditions (musculoskeletal, cardiovascular, pulmonary, neurologic) that incapacitate the patients from performing all the motor and cognitive tests.

Patients participated in T1 measurements under the condition of a negative COVID-19 test, which was performed when either of the following conditions was met: on the day of hospital discharge or the 10th day after confirmation of the disease, if they had been discharged from the hospital earlier. The time frame for recruiting patients for T1 measurements was between January 7th and March 18th, 2021. After the T1 assessment, they received a brochure “Stay active” (30) with general information about the beneficial effects of physical activity (PA) and some comprehensive explanation of how different exercises could be performed at home during the COVID-19 pandemic. This was an invitation to engage in rehabilitation activities. However, adherence to these recommendations was not monitored.

This study was conducted according to the guidelines laid down in the Declaration of Helsinki, and all procedures involving research study participants were approved by the institutional ethical board of the Izola General Hospital (application number: 1/21, approved February 1, 2021). All patients were evaluated by a dedicated physician during their hospital stay, during which the study protocol was thoroughly explained. Written informed consent was obtained from each participant before any assessments, contingent upon their agreement to participate in the study. The clinical trial protocol has been registered on ClinicalTrials.gov with the identifier number NCT04860206.

### 2.2 Measurements

All assessments at T1 and T2 times were performed by a trained researcher in the same room, using the same equipment. Measurements were performed in a standardized order. Body mass (kg) and height (m) were measured using a calibrated scale (Libela-Elsi Ltd., Slovenia), with values rounded to the nearest 0.1 kg and 0.5 cm.

The body composition was measured using the tetrapolar bioimpedance device BIA 101 Anniversary (Akern-Srl, Florence, Italy), after participants were lying supine for 30 min. The proportion of fat mass (FM in kg and %) and fat-free mass (FFM in kg and %) was recorded. The fat mass-to-fat-free mass ratio (FM/FFM ratio) was defined as the total fat mass divided by the total fat-free mass, providing an index of the proportion of adipose tissue relative to fat-free mass.

Adherence to the Mediterranean lifestyle was assessed using the MEDLIFE index questionnaire (31). The Mediterranean Lifestyle (MEDLIFE) index is a validated tool designed to evaluate an individual’s adherence to dietary and lifestyle principles characteristic of the Mediterranean region (31). A total of 28 items are divided into three blocks of questions (food consumption frequency—section 1, Mediterranean dietary habits—section 2, and Mediterranean lifestyle—section 3). For each item, 1 point is given (in total, 28 points) if the answer meets certain criteria. The total score ranges from 0 to 28, with higher scores indicating greater adherence to the Mediterranean lifestyle.

### 2.3 Qualitative assessments

Structured interviews and questionnaires were used to assess participants’ perceived physical, mental, and emotional responses to COVID-19. This study did not employ a focus group design; rather, individual structured interviews were conducted with selected participants. Interviews were conducted following functional assessments by the researcher. Interviews were recorded in written form during data collection. As part of a broader assessment protocol, interviews were intentionally brief and structured (approximately 10 min) to avoid interfering with clinical measurements. Responses were recorded immediately to ensure accuracy. At T1, the interview focused on patients’ subjective experience of hospitalization due to COVID-19. Specifically, patients were asked what they found most distressing aside from physical symptoms, what coping strategies they employed, and what they missed most during their hospital stay. They also re-evaluated their perceived physical and mental state (as a percentage) after the infection, using their pre-infection state as a 100% baseline. At the end of the interview, patients were provided with a tracking form to monitor symptoms and well-being, which they were asked to bring to the follow-up appointment after the rehabilitation. Additionally, they received a “Stay home - Be active” manual, which included exercises tailored for individuals with chronic respiratory disease. At T2, patients were asked to reassess their physical condition and mental state, and mood, using their pre-infection state—assumed to represent 100%—as a reference point. They were also invited to reflect on the specific challenges encountered during recovery, including experiences of frustration, thoughts that arose during their recovery, coping strategies employed, and any perceived gaps in support or resources that might have facilitated a more successful recovery. Participants were also asked to identify what they believed was necessary to reach 100% recovery across physical, mental and mood state, and to estimate when they expect to reach that goal. Finally, patients were asked whether they had followed the guidance provided in the ‘Stay Home—Be Active’ manual and if they had engaged in the recommended exercises.

This qualitative approach enabled the integration of quantitative physiological outcomes with qualitative insights into patients’ lived experiences, providing a more comprehensive understanding of recovery processes.

### 2.4 Data analyses

All statistical analyses were performed with the statistical software IBM SPSS 27.0 (IBM Corp, Armonk, NY, USA). Data are presented as mean values and standard deviations (SD). The normality of the distribution was checked and confirmed both graphically (using histograms and QQ plots) and analytically (via the Kolmogorov–Smirnov test). To examine changes in outcomes from hospital discharge to post-rehabilitation, a repeated-measures analysis of covariance (RM-ANCOVA) was conducted, with time (T1 vs. T2) as the within-subject factor and sex included as a covariate to control for its potential confounding effect. To examine the associations between body composition parameters and adherence to the Mediterranean diet, Spearman’s correlation coefficient was applied for body composition parameters and Sections 1, 2, 3, and the overall MEDLIFE index score. A *p*-value < 0.05 was considered statistically significant.

For each of the three MEDLIFE sections and the overall MEDLIFE index, we calculated z-scores. We identified the top 25% of individuals (i.e., those in the highest quartile based on the overall MEDLIFE z-score). To ensure consistency in our analysis and focus on adherence primarily driven by diet and nutrition, we included only participants who fell within the top quartile (Q4) in Section 1 (Mediterranean food consumption), Section 2 (Mediterranean dietary habits), and the overall MEDLIFE index. This approach excluded individuals who scored highly solely due to lifestyle-related behaviors captured in Section 3 (physical activity, social habits, and conviviality), as these did not always overlap with high scorers in Sections 1 and 2. By doing so, we focused our analysis on individuals who adhered more closely to the Mediterranean dietary pattern. Qualitative data were analyzed using a thematic analysis approach based on a predefined coding framework aligned with the interview structure. The basic nodes for analysis related to the rehabilitation period included: physical challenges, cognitive strain, emotional responses (e.g., frustration), thought processes during rehabilitation, types of support received, aspects missed during rehab, assessments of full recovery, recovery timelines and cross-cutting patterns.

Given the exploratory nature of the study and the clinical setting, analyses were interpreted with consideration of the sample size.

## 3 Results

The general characteristics of patients who participated in the study are described in Table 1 for T1 and T2. At T2, fat-free mass significantly increased (*F* = 15.370, *p* < 0.001, η^2^_p_ = 0.293).

**Table 1:** Clinical and body composition characteristics of participants at hospital discharge (T1) and after 2 months of rehabilitation (T2), controlled for sex.

Variables	T1	T2	*F* (*p*)
Age (years)	59.0 ± 10.3	/	/
Body height (cm)	175.9 ± 8.7		
Former smoker, n (%)	6 (15.4)		
Current smoker, n (%)	1 (2.6)		
Comorbidities, n (%)	7.9 ± 1.6		
Body mass (kg)	98.9 ± 16.6	98.7 ± 16.3	0.335 (0.567)
Fat mass (%)	30.5 ± 7.5	29.2 ± 8.7	0.269 (0.607)
Fat-free mass (%)	69.6 ± 7.4	70.8 ± 8.7	15.370 (<0.001)
FM/FFM ratio	0.45 ± 0.17	0.43 ± 0.19	0.364 (0.550)
MEDLIFE index—total	25.8 ± 6.4	24.3 ± 6.8	0.139 (0.711)
MEDLIFE index—section 1	6.8 ± 2.4	6.3 ± 2.6	0.384 (0.539)
MEDLIFE index—section 2	4.3 ± 1.7	4.1 ± 1.7	0.006 (0.939)
MEDLIFE index—section 3	3.2 ± 1.3	2.9 ± 1.4	0.023 (0.881)

FM/FFM, fat mass vs. fat-free mass ratio.

**Figure 1:**
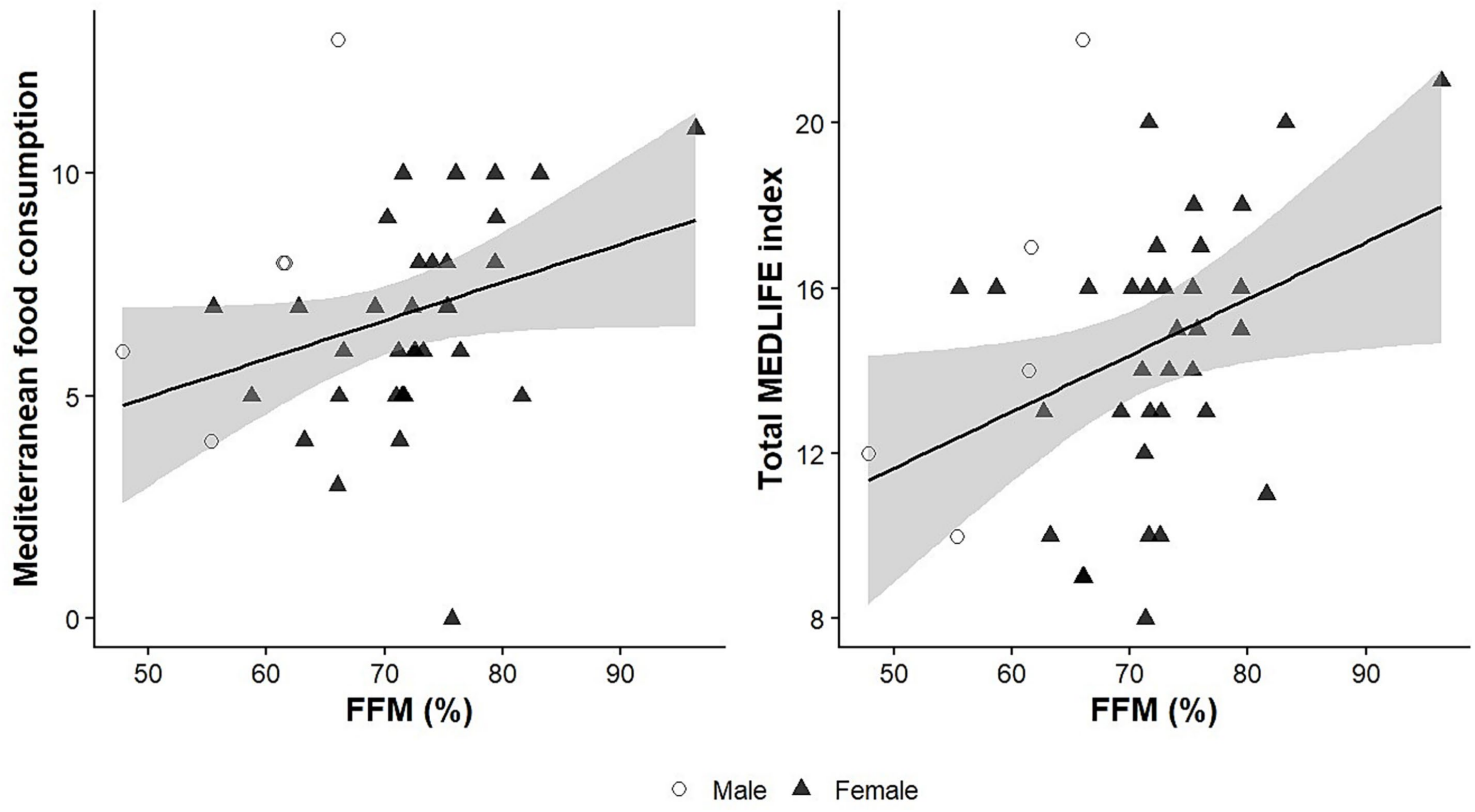
Associations between fat-free mass (FFM, %) and (A) Mediterranean food consumption (MEDLIFE section 1) and (B) total MEDLIFE index score at follow-up (T2). Solid lines represent linear regression fits, with shaded areas indicating 95% confidence intervals. Circles represent male participants and triangles represent female participants.

There was a positive correlation between fat-free mass at T2 and adherence to section 1 of the MEDLIFE index (Mediterranean food consumption), and a positive correlation between fat-free mass at T2 and total Mediterranean adherence (r = 0.337, *p* = 0.036; r = 0.319, *p* = 0.047) (Figure 1).

For further data analysis, participants who demonstrated the highest adherence to the Mediterranean lifestyle or its individual components are presented in Table 2. With those participants, a more detailed examination of their interviews, that is, the fourth quartile of participants (Q4), was conducted.

**Table 2:** MEDLIFE index scores of participants in the highest adherence quartile (Q4).

ID	MEDLIFE index-total (max = 28 pt)	MEDLIFE index-part 1 (max = 15 pt)	MEDLIFE index-part 2 (max = 7 pt)
PC1008	20	10	7
PC1011	21	11	6
PC1013	22	13	6
PC1021	20	10	6
PC1036	18	9	6

ID, identification number; pt, points; Q4, participants in the highest quartile of adherence to the Mediterranean lifestyle based on the overall MEDLIFE index score; Q1–Q3, participants in the lower three quartiles.

A focused qualitative analysis was conducted on the Q4 group (n = 5), with particular attention given to their adaptive responses throughout an eight-week rehabilitation program. To identify potential differences, their qualitative data were systematically compared to those of participants with lower outcomes on the MEDLIFE Index, group Q1-Q3 (n = 34). Given the limited representation of female participants (less than 20% of the total sample), gender was not treated as a comparative variable in the analysis.

Comparison of qualitative analysis of post-recovery experience between Q4 and Q1-Q3 groups is presented in Table 3. The comparative framework concentrated on several core dimensions: self-reported physical, cognitive, and emotional functioning; coping mechanisms adopted during the rehabilitation process; perceived deficits in support or resources during recovery; and subjective evaluations of rehabilitation success. The primary objective was to discern both objective and phenomenological distinctions between groups that may be associated with observed differences in rehabilitation outcomes.

**Table 3:** Comparison of recovery experiences and coping strategies between high (Q4) and lower (Q1–Q3) adherence groups.

Dimension	Q4	Q1–Q3	Key differences
1. Physical difficulties	Climbing stairs, general weakness, breathing during effort,	Fatigue, leg weakness, muscle pain, reduced stamina, difficulty walking/lifting.	Q4 reported more localised issues; Q1-Q3 described more extensive physiological problems.
2. Mental effort post-illness	Mostly no issues, a few noticed memory/concentration problems.	Widespread cognitive fatigue, memory loss, and focus issues.	Q1-Q3 experienced greater cognitive load and mental strain.
3. Emotional challenges	Mild or no frustration; some emotional reflection (e.g., concern over lab results).	More frequent frustration, anxiety, and fear of permanent damage or reinfection.	Q1-Q3 displayed higher emotional distress and frustration than Q4.
4. Thoughts during rehabilitation	Dominantly positive, focused on growth, acceptance, and routine changes.	Mixed: some positive, but also characterised by passivity and resignation.	Q4 showed more proactive coping; Q1-Q3 had more emotional withdrawal.
5. Support and suggestions	Trusted individuals, physiotherapy, and structured physical activity.	Institutional rehab, psychological care, family interaction, and group motivation.	Q1-Q3 desired more external structure and emotional support. Q4 relied on routine and self-help.
6. Missed during rehab	Nothing major or just a lack of professional oversight.	Missed human connection, freedom of movement, and organised rehab.	Q1-Q3 reported greater social isolation and lack of formalised care.
7. Perceived 100% recovery	Most were already at or close to 100% in all domains.	Physical is often 100%, but mental/emotional lags behind.	Q4 recovered faster and more evenly across all areas.
8. Time to full recovery	Max. 2–3 months or already there.	Physical: 1–3 months; Mental/Emotional: 3–6 + months, often unclear.	Q1-Q3 had a slower and more uncertain recovery in mental/emotional domains.

Q4, participants in the highest quartile of adherence to the Mediterranean lifestyle based on the overall MEDLIFE index score; Q1–Q3, participants in the lower three quartiles.

A subsequent descriptive analysis was performed to enhance the clarity and interpretability of potential intergroup differences, with the objective of elucidating patterns that may not have been fully captured through qualitative comparison alone (Table 4).

**Table 4:** Summary of key differences in recovery outcomes between high (Q4) and lower (Q1-Q3) adherence groups.

Category	Q4	Q1–Q3
Physical difficulties	4/5 reported mild fatigue	~70% reported fatigue, breathing
Mental strain	3/5 no issues	~60% with memory/focus issues
Frustration	3/5 no frustration	~55% with frustration
Positive mindset during rehab	5/5	~50%
Missed rehab support	2/5 said nothing was missed	~65% missed structure/social contact
100% physical recovery	5/5	~75–80%
100% mental recovery	5/5 or soon	~50% uncertain
100% emotional recovery	5/5 or soon	~40–50%, often delayed

Q4, participants in the highest quartile of adherence to the Mediterranean lifestyle based on the overall MEDLIFE index score; Q1–Q3, participants in the lower three quartiles.

Participants in Q4 exhibited higher levels of resilience and optimism throughout their recovery process. They reported comparatively faster physical and emotional improvements, lower levels of frustration, and greater self-regulation during rehabilitation. The majority of individuals in this group anticipated or had achieved full recovery within two to three months. Their recovery strategies were characterized by adherence to personal routine, acceptance of their condition, and limited reliance on external support systems. These findings are further supported by participants’ own accounts, which provide insight into the coping strategies they employed to manage their recovery and support themselves throughout the process:

*“With a smartphone and sudoku, I was calming myself and enjoyed the food. Yes, the hospital food was good./I kept thinking positively all the time, and from the critical point onward, I kept getting better."(PC1008 from Q4)*

*“I believed everything would turn out well, as everyone was sending me positive energy." Regarding the strategies they used or what had helped them, one participant explained: ‘It helped me calm down; I have structure in my day — I eat breakfast, have lunch, and prepare my meals myself.” (PC1013 from Q4)*

In contrast, participants in Q1-Q3 encountered more complex challenges that spanned physical, emotional, and cognitive domains. These participants frequently struggled with uncertainty about their recovery, expressed heightened frustration, and experienced significant social isolation.

*“I felt powerless — even taking a shower was exhausting. I was afraid of the final consequences, afraid of what might happen because of my lung problems.” (PC1014)*

*” You try to stay calm, even though you are upset and sad… I followed the doctor’s instructions, received the antibiotic infusion, practiced the breathing exercises and endured the pain while waiting for things to get better.” (PC1023)*

Their recovery trajectories, particularly in terms of mental and emotional well-being, were slower, less predictable, and often still ongoing. Unlike Q4, individuals in Q1-Q3 showed a greater need for institutional support, structured care programs, and guided rehabilitation to manage the lingering effects of illness.

*“I am angry that there was no guided rehabilitation; we should have undergone rehabilitation in a spa, under medical supervision.” (PC1007)*

*“To have the possibility of joining a group with similar difficulties, participating in professionally guided physical exercise, and receiving cognitive guidance.” (PC1028)*

## 4 Discussion

This study aimed to investigate the association between adherence to the Mediterranean diet, body composition characteristics, and recovery-related health outcomes in patients who have recovered from severe COVID-19. Since this study was conducted, other papers have been published examining multiple aspects of post-COVID-19 recovery, including physiological and cognitive outcomes (32, 33). In this study, an explanatory mixed-methods design (34) was employed. In the first phase, quantitative analyses of body composition were conducted, while in the second phase, qualitative analysis of structured interviews and questionnaires provided a more comprehensive understanding of adherence to the Mediterranean diet.

Results showed that fat-free mass increased after rehabilitation (*p* < 0.001). Further analysis of body composition parameters revealed a positive correlation between fat-free mass and Section 1 of the MEDLIFE index (*p* = 0.037) as well as with the total MEDLIFE index score (*p* = 0.047). Section 1 assesses the frequency of consumption of key food groups characteristic of the Mediterranean dietary pattern. Its purpose is to evaluate the extent to which an individual’s dietary habits align with the core principles of the traditional Mediterranean diet, which is predominantly plant-based and characterized by high intake of fruits, vegetables, legumes, whole grains, and healthy fats—particularly olive oil—together with moderate consumption of animal-derived products and limited intake of processed foods and added sugars (35). These dietary components are known to support immune function and reduce systemic inflammation and may be associated with a more robust physiological response during and after COVID-19 infection (27). Moreover, higher consumption of fruits and vegetables, rich in polyphenols and micronutrients, may help preserve fat-free mass through indirect mechanisms involving the reduction of oxidative stress (36). Although the associations identified in our study are cross-sectional and cannot establish causality, it can be suggested that specific dietary components captured in Section 1 of the MEDLIFE index may be associated with favourable body composition outcomes. Previous literature has already established a positive association between body composition parameters, including FFM, and adherence to the Mediterranean diet (35–37). These findings underscore the importance of dietary quality—not just quantity—as a modifiable determinant of muscle health (37), particularly in populations at risk of muscle mass decline. Furthermore, the total MEDLIFE index score, reflecting overall adherence to the Mediterranean lifestyle, was positively associated with FFM, suggesting that individuals with greater adherence to Mediterranean lifestyle principles are more likely to preserve higher levels of muscle mass. Higher adherence to the Mediterranean lifestyle, which encompasses not only dietary quality but also physical activity and social habits, may be associated with the preservation of muscle mass and the maintenance of overall health during a period marked by reduced mobility, disrupted daily routines, and increased vulnerability to infection-related complications (38, 39). The Mediterranean lifestyle promotes the consumption of nutrient-dense, anti-inflammatory foods, alongside regular physical activity, both of which are considered important for maintenance of FFM. FFM is not only an indicator of physical strength and metabolic health, but also plays a pivotal role in immune competence (40). Preservation of FFM has been linked to improved outcomes during infections and a reduced risk of complications, a consideration of particular relevance in the context of COVID-19, where muscle loss and general deconditioning were prevalent due to prolonged inactivity or post-infection recovery (41). Given that FFM is a critical determinant of resilience during illness, especially viral infections such as COVID-19, these observations highlight the importance of promoting Mediterranean dietary habits as part of public health strategies aimed at enhancing population health and preparedness for future pandemics. Interpretative Phenomenological Analysis (33), combined with descriptive analysis, was employed to examine participants’ subjective experiences of hospitalization due to COVID-19. This approach, which emphasizes individuals’ perceptions of lived experiences, facilitates the generation of contextually grounded insights into psychological and behavioural responses to hospitalization and recovery. The method allowed for a nuanced and interpretive understanding of COVID-19 as a significant life event.

Further qualitative analysis was undertaken for the final quartile of participants (Q4)—those demonstrating the highest adherence to the Mediterranean diet and lifestyle—to obtain a deeper understanding of their lifestyle characteristics and coping strategies. The findings observed from this subgroup corresponded closely with the broader framework of health-related recovery and resilience. Participants in the Q4 group display notable resilience, which appears to be strongly associated with consistent daily routines and a sustained commitment to self-care, particularly in relation to nutrition and personal growth. These findings align with the core dimensions of recovery and long-term health, which emphasize sustained, long-term engagement in health-promoting behaviours and proactive self-regulation across the lifespan. Most participants in the Q4 group appeared to have internalized the principles of the Mediterranean lifestyle, consistent with previous evidence indicating that adherence to the Mediterranean diet and related lifestyle practices enhances both physiological and psychological resilience to illness (24, 42, 43). The COVID-19 pandemic offered a real-world context in which these protective factors were distinctly evident. The present findings further highlight the potential role of Mediterranean lifestyle adherence in supporting health, well-being, and adaptive capacity under conditions of heightened adversity.

### 4.1 Strengths and limitations

This study offers a comprehensive and patient-centred perspective on the recovery of individuals who have suffered a severe COVID-19 illness, examining both their dietary patterns, particularly adherence to the Mediterranean diet, and their life experiences through first-person interviews. A major strength of this approach is the combination of quantitative and qualitative methods, which allows for a deeper understanding of not only dietary habits but also subjective challenges and perceptions of recovery. The focus on a high-risk population increases the clinical relevance of the results, and the inclusion of personal narratives provides valuable insights into the psychological, behavioural, and social dimensions of life after COVID-19 disease that are often overlooked in purely clinical studies.

However, several limitations should be acknowledged. First, the study relies on self-reported dietary data and retrospective reports, which are susceptible to recall bias and social desirability effects. Second, the relatively small qualitative sample size constrains the generalizability of the findings and may limit the achievement of full thematic saturation. Due to the exploratory nature of the study and the clinical constraints during the COVID-19 pandemic, no formal power analysis was conducted, and the sample size may limit statistical power. Furthermore, the absence of baseline data or a control group limits the ability to draw causal inferences or to assess changes over time. The predominance of male participants limits generalizability, particularly to female populations. Moreover, due to the limited number of female participants, potential sex-specific differences and interactions between sex and dietary adherence could not be reliably assessed. Potential confounding factors, including gender, comorbidities, socioeconomic status and variation in rehabilitation support, may influence both dietary behaviours and recovery outcomes. Physical activity was assessed only at follow-up and not at both time points; therefore, its potential contribution to the observed changes in fat-free mass could not be evaluated longitudinally and should be considered a possible confounding factor.

Despite these limitations, the study offers valuable exploratory insights and underscores the importance of integrating nutritional and patient-reported perspectives into post-COVID-19 rehabilitation strategies.

## 5 Conclusion

This article highlights the Mediterranean diet as a sustainable and culturally adaptable approach to supporting recovery, enhancing quality of life, and promoting recovery and long-term health in post-COVID-19 rehabilitation. Moreover, it explores how adherence to the Mediterranean diet relates to body composition and recovery-related indicators in individuals recovering from severe COVID-19. It suggests that higher adherence to the Mediterranean diet may be associated with greater resilience and more favourable body composition within the broader context of healthy ageing, particularly in response to viral infections such as COVID-19. The recovery patterns observed among the group of higher adherences to the Mediterranean diet (Q4) indicate that key elements of recovery and long-term health—lifestyle management, social connectivity, and cognitive engagement—may be associated with reduced physical and psychological challenges during convalescence. Their reliance on structured routines, emphasis on prevention, and active coping strategies exemplify key mechanisms of the recovery and resilience framework, which also includes community support, age-friendly environments, and digital inclusion. Accordingly, the Q4 group provides insights into how health systems could consider integrating recovery and long-term health principles through integrated, person-centred approaches spanning medical, behavioural, and social domains.

## Data Availability

The raw data supporting the conclusions of this article will be made available by the authors, without undue reservation.
